# Effects of co-adsorption on interfacial charge transfer in a quantum dot@dye composite

**DOI:** 10.1186/s11671-021-03604-0

**Published:** 2021-09-20

**Authors:** Peng Cui, Yuan Xue

**Affiliations:** Nanotechnology Research Laboratory, School of Textile Science and Engineering, Jiangnan University, No.1800 Lihu Road, Wuxi, 214122 Jiangsu Province People’s Republic of China

**Keywords:** Nanocrystals, Dye, Interfacial charge separation, Electronic coupling, Energy alignment, Solar energy conversion

## Abstract

The sensitive electronic environment at the quantum dot (QD)–dye interface becomes a roadblock to enhancing the energy conversion efficiency of dye-functionalized quantum dots (QDs). Energy alignments and electronic couplings are the critical factors governing the directions and rates of different charge transfer pathways at the interface, which are tunable by changing the specific linkage groups that connect a dye to the QD surface. The variation of specific anchors changes the binding configurations of a dye on the QD surface. In addition, the presence of a co-adsorbent changes the dipole–dipole and electronic interactions between a QD and a dye, resulting in different electronic environments at the interface. In the present work, we performed density functional theory (DFT)-based calculations to study the different binding configurations of N719 dye on the surface of a Cd_33_Se_33_ QD with a co-adsorbent D131 dye. The results revealed that the electronic couplings for electron transfer were greater than for hole transfer when the structure involved isocyanate groups as anchors. Such strong electronic couplings significantly stabilize the occupied states of the dye, pushing them deep inside the valence band of the QD and making hole transfer in these structures thermodynamically unfavourable. When carboxylates were involved as anchors, the electronic couplings for hole transfer were comparable to electron transfer, implying efficient charge separation at the QD–dye interface and reduced electron–hole recombination within the QD. We also found that the electronic couplings for electron transfer were larger than those for back electron transfer, suggesting efficient charge separation in photoexcited QDs. Overall, the current computational study reveals some fundamental aspects of the relationship between the interfacial charge transfer for QD@dye composites and their morphologies which benefit the design of QD-based nanomaterials for photovoltaic applications.

**Supplementary Information:**

The online version contains supplementary material available at 10.1186/s11671-021-03604-0.

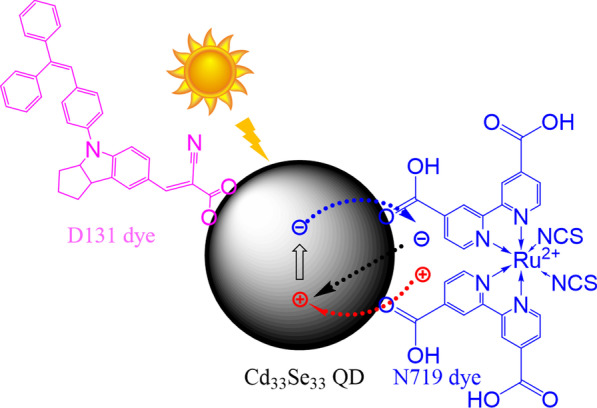

## Introduction

Dye-sensitized photocatalytic water splitting or photovoltaic systems have stimulated intense interest in the research community because of their potential to become a substitute for non-renewable energy resources. The dye molecules are ideal catalytic agents for oxidation or reduction reactions due to long-lived metal-to-ligand (MLCT) states [1]; for example, $\left[ {{\text{Ru}}\left( {{\text{bpy}}} \right)_{3} } \right]^{2 + }$ and many of its derivatives have a lifetime of approximately 600 ns. In addition, the ultrafast charge injection from the dye to the semiconductor and slow electron recombination from the semiconductor to the dye promise efficient charge separation at the interface, a key factor governing the solar-to-electrical energy conversion. However, the dye molecule only absorbs limited wavelengths of light, making it less efficient in managing these processes.

Quantum dots (QDs) possess size-tunable electronic and optical properties [2]; therefore, they can convert solar energy more efficiently than dyes. In particular, the multi-carrier generations [3] significantly boost the number of charge carriers generated by absorbing photon energies. Furthermore, in the strong quantum confinement region, electrons and holes are treated approximately as independent particles. Therefore, their coulomb interactions are negligible compared to the quantization effect, which reduces the electron–hole recombination rates in QDs. This allows more photogenerated charge carriers to participate in a photocatalytic reaction or photovoltaic energy conversion process.

Upon the photoexcitation of QDs, the electrons are promoted from the valence band (VB) to the conduction band (CB) of the QDs. An idealized photovoltaic or photochemical cell is expected to achieve efficient separation of photoexcited electron–hole pairs across the QD and dye interface. On the one hand, this increases the lifetimes of charge carriers in QDs; on the other hand, it changes the dye’s oxidation states, allowing it to work as an oxidizer or reducer. For example, Mora-Seró et al. fabricated a cadmium selenide (CdSe) QD-sensitized solar cell where the dye molecules were placed on the QD surface to extract holes from the VB edge of the QD, which reduces the electron–hole recombination and increases photocurrents in QDs [4]. Gimbert-Suriñach et al. used cadmium telluride (CdTe) QDs as light harvesters to transfer photoexcited electrons to a cobalt catalyst, which catalyses the hydrogen evolution reaction [5].

Despite the great promise of using QD@dye composites as light sensitizers, their efficiencies are relatively low. This is likely because the specific type of dye attached to the QD surface changes the electronic environment at the QD–dye interface, namely the interfacial effect. The interfacial effect is caused by the QD–dye interaction that modifies their electronic and geometrical structures at the region where the two molecules make contact. In particular, the dye can be bound to the QD surface with different orientations by controlling the deprotonation by varying PH environments. This alters the electronic and optical properties of QD@dye composite, depending on the binding conformations of dye. For example, the deprotonation of carboxylic acid functionalized ligand of dye results in a shift of photoluminescence emission spectra. Varying the binding geometry of dye on the QD surface changes the energy alignment of the QD and the dye at the occupied and unoccupied levels, thereby changing the charge transfer direction. Furthermore, depending on the electronic coupling between the two molecules, it changes how fast the photoexcited charge carrier is delivered from the QD to the dye or vice versa. These two factors, that is, the direction of charge transfer and the electronic coupling, are a complex interplay of the QD size [6, 7], QD composition [4], structure of the dye [8, 9], QD–dye interaction [10, 11], and solvent environment [12, 13]. Unfortunately, it is a difficult task to probe these properties at the QD–dye interface through experimental means due to a lack of signatures of these properties in conventional spectroscopy techniques.

Experimental approaches [14–16] and theoretical computations [17] can allow for direct observations of a dye’s morphologies on a semiconductor surface [18]. However, only a few studies report on the binding of multiple dyes to semiconductor surfaces, with the majority of studies focusing on binding a single dye to a semiconductor surface [10, 11, 19]. In addition, despite a large number of studies focused on the binding configurations of dye molecules to a titanium dioxide (TiO_2_) surface, very little attention has been given to CdSe QDs [11], cadmium sulphide (CdS) QDs [20], and CdTe QDs [21]. Detailed systematic studies on the effect of the adsorption geometries of dyes on the electronic structures of QDs are needed. In previous work [11], we studied a single Ru(II)-polypyridyl complex (i.e. N719 dye) attached to the surface of a CdSe QD and the related charge transfer properties using density functional theory (DFT)-based calculations. Our results suggested that the relative positions of the dye and QD’s orbitals were fairly sensitive to the adsorption geometries of the N719 dye on the CdSe QD surface, controlled through the specific linkage group anchoring the dye to the QD surface. However, our previous work only considered the attachment of a single dye to the QD surface. In contrast, in practice, multiple dye molecules of different types might be attached to the QD surface [8, 22]. Accordingly, this changes the QD–dye interaction and, thereby, the charge separation dynamics at the QD–dye interface compared to the case of the attachment of a single dye. Unravelling such details offers new insights into the performance of photovoltaic and photochemical devices based on QD@dye composites concerning their morphologies.

In the current work, we performed DFT-based calculations to study the electronic and geometrical structures of a CdSe QD functionalized with N719 dye and a co-adsorbent, that is, D131 dye. N719 dye and its derivatives consistently show a high performance among many photosensitizers developed for photovoltaic and photocatalytic cells. However, it is challenging to determine the atomic-scale structure of the N719–semiconductor interface. Our previous work [11] suggested several possible binding configurations of N719 dye on the CdSe QD surface and raised some fundamental issues; for example, the effect of multiple dye adsorption on the charge transfer between QDs and dyes is still not fully understood. Therefore, we continued to investigate the adsorption stability of N719 dye on the CdSe QD surface along with a co-adsorbent, D131 dye. D131 dye is typically used to expand the absorption spectrum of primary adsorbent dyes and avoid their aggregation in dye-sensitized solar cells [23]. We investigated how the co-adsorption of D131 dye affected the adsorption stability of N719 dye on the CdSe QD surface. In addition, we studied the effect of co-adsorption on the electronic couplings of different binding configurations.

## Computational methodology

The ground-state geometries of the N719-D131 dye@Cd_33_Se_33_ QD composites were obtained from a DFT geometry optimization with the B3LYP functional and LANL2DZ/6-31g* mixed basis set. LANL2DZ was applied for the transition metal with relativistic corrections, and 6-31 g* was used for the non-metal elements. Hybrid-GGA functionals with a mixed basis set have commonly been used for simulating hybrid organic–inorganic systems [24]. The simulated optical spectra of such systems correspond well with the experimental results [25–27]. The solvent effect was included using the polarizable continuum model. After the geometry optimization, the total density of states (TDOS) and contribution of the specific molecular component to the TDOS, that is, PDOS, were calculated as in Eq. 1 [11]:

1 $${\text{PDOS}}\left( \varepsilon \right) = \frac{1}{\tau \sqrt \pi }\mathop \sum \limits_{n} w_{n} \exp \frac{{ - \left( {\varepsilon_{n} - \varepsilon } \right)^{2} }}{{\tau^{2} }},$$

where ${\varepsilon }_{n}$ is the nth Kohn–Sham energy, ${w}_{n}$ is the weight of the nth Kohn–Sham orbital from the specified molecular component, and $\tau$ is the line broadening parameter of 100 meV [11] to account for the thermal fluctuations of atoms. For the TDOS, ${w}_{n}=1$.

**Fig. 1:**
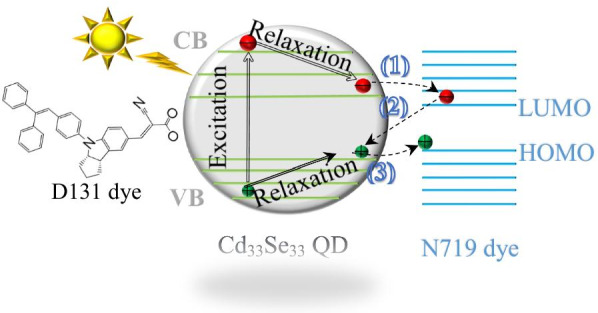
Illustration of different charge transfer pathways upon the photoexcitation of the Cd_33_Se_33_ QD functionalized with N719-D131 dyes: (1) electron transfer (***et***) from the CB edge of the QD to the LUMO of the dye, (2) electron back transfer from the LUMO of the dye to the QD VB edge (***re***), and (3) hole transfer (***ht***) from the QD VB edge to the HOMO of the dye

We considered three charge transfer pathways at the interface, as illustrated in Fig. 1: (1) QD-to-dye electron transfer (***et***), (2) dye-to-QD electron–hole recombination (***re***), and (3) QD-to-dye hole transfer (***ht***). These processes occur with electrons or holes located at the lowest unoccupied orbitals or highest occupied orbitals of QDs or dyes, as suggested from previous experimental [4, 28] and theoretical works [29, 30], and determine the overall charge separation efficiency at the QD–dye interface. The equilibrium geometries of these charge transfer states were optimized based on the constrained DFT (CDFT) method with density constraints [11, 31–33] to obtain the charge-localized diabetic reactant and product states. Afterwards, the electronic coupling was obtained by solving the secular equation, as implemented in the NWChem 6.8 software package [34]. The same functional and basis set were employed throughout all the calculations.

## Results and discussion

### Electronic and geometrical structures of QD@dye composites

**Scheme 1:**
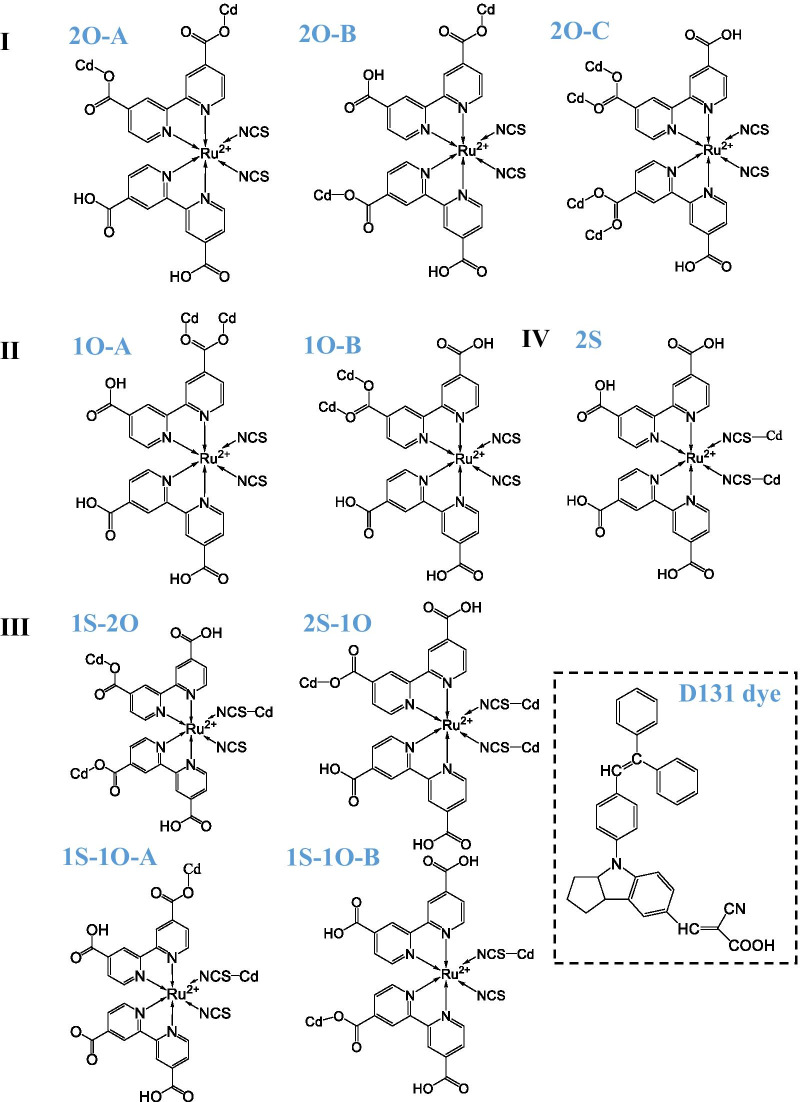
The different binding configurations of N719 dye attached to the Cd_33_Se_33_ QD can be summarized into four categories. The schematic structure of D131 dye is shown in the dashed line box. Each category contains different binding configurations of the dye, distinguished by A, B, and C

**Fig. 2:**
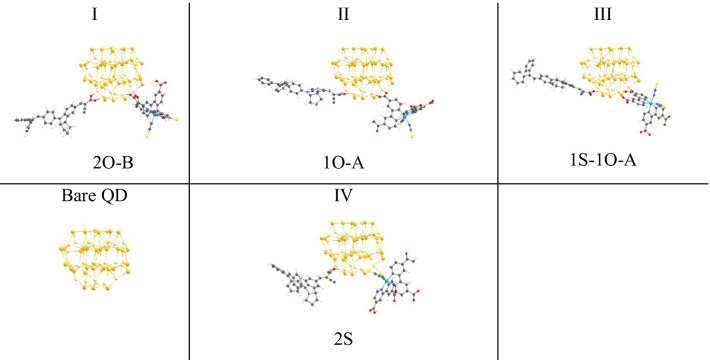
Representative geometries of the bare Cd_33_Se_33_ QD and N719-D131@Cd_33_Se_33_ composite. The geometry optimization was performed in vacuum

N719 and D131 dyes were connected to the Cd_33_Se_33_ QD surface through the most reactive site, that is, the site where all the surface cadmium (Cd) atoms were 2-coordinated. The attachment of N719 dye to the QD surface can be summarized in four categories depending on the position of carboxylate anchor in the bipyridine ligand of dye. In each category, the binding geometry varied concerning the position of carboxylate in the bipyridine ligand, as shown in Scheme 1. The three-dimensional views of these structures are provided in Fig. 2 and Additional file 1: Fig. S1. In our notation system, the “O” and “S” represent the carboxylate and isocyanate anchors, respectively. The number indicates how many anchors are used to connect the dye to the QD surface. The following letter indicates different deprotonation sites. Changing the number of deprotonated carboxyl groups changes the binding sites and surface orientation of the N719 dye. Previous studies have shown that the binding modes of Ru(II) complexes are sensitive to the pH environment at the semiconductor–dye interface [17, 35, 36]. The XPS showed different “O” binding energies associated with different binding geometries of Ru(II) complexes on the semiconductor surface [37]. Therefore, our findings are consistent with previous studies.

Based on previous theoretical and experimental studies, we did not include the surface ligands in the current model since they have an insignificant impact on the electronic structure of colloidal QDs [38, 39]. Therefore, the reduced QD@dye model without ligand passivation serves as an idealized tool to study the atomic details of the QD–dye interface with affordable computational costs. Another study has further justified this by showing that attaching a single Ru(II) complex to a CdSe/ZnS QD is experimentally possible, which creates mid-gap states that quench the fluorescence of QDs [40].

The most common experimental size for CdSe QDs is approximately between 2 and 4 nm; however, the qualitative trend of QD–dye interactions is expected to be insensitive to the QD size. Increasing the QD size only increases the number of Cd atoms on the surface, while the lattice symmetries of different facets and edges are barely affected at the low concentrations of the dyes [11, 41].

Table 1 shows that the overall binding energies between the N719 dye and Cd_33_Se_33_ QD in N719-D131@Cd_33_Se_33_ composites are approximately between 1.42 and 2.83 eV, which are lower than those of Cd_33_Se_33_ QDs functionalized with a single N719 dye, as shown in Additional file 1: Table S1. The lower binding energies are consistent with the longer Cd–O bond lengths in these structures. Our result is similar to Honda et al.’s experimental observation that the D131 dye reduces the number of binding configurations of the N719 dye on the semiconductor surface [22]. In their case, the isocyanate anchors of the N719 dye were completely detached from the surface of TiO_2_ anatase due to the co-adsorption of D131 dye [22]. The most stable adsorption geometries of N719 dye involve two carboxylate groups as anchors, as indicated by the highest binding energies in Category I. This is consistent with experimental findings that double carboxylate anchors provide dye molecules with the most stable adsorption configurations on the semiconductor surface [42, 43]. Increasing the number of isocyanate anchors increases the Cd–S bond lengths accompanied by a decrease in the binding energies; for example, the Cd–S bond length in 1S–2O is 2.73 Å compared to 2.84 Å in 2S–1O. However, the Cd–O bond length shows no significant dependence on the number of deprotonated carboxyl groups in Categories I and II. The inclusion of isocyanate anchors reduces the binding stability of the dye molecules on the QD surface. For example, the binding energies of those structures involving isocyanate groups as anchors (Categories III and IV) are lower than those involving carboxylate groups as anchors (Categories I and II).

**Table 1:** Electronic and geometrical properties of the N719-D131@Cd_33_Se_33_ composite. The geometries were optimized in vacuum

Binding configuration	Binding energy (eV)	Band gap (eV)	Cd–O bond (Å)	Cd–S bond (Å)
I				
2O-A	− 2.83	0.76	2.36	
2O-B	− 4.32	1.46	2.37	
2O-C	− 4.46	1.51	2.34	
II				
1O-A	− 2.19	1.37	2.42	
1O-B	− 2.27	1.41	2.39	
III				
1S–2O	− 2.18	1.66	2.26	2.73
2S–1O	− 1.44	1.36	2.30	2.84
1S–1O-A	− 1.97	1.52	2.42	2.73
1S–1O-B	− 2.06	1.14	2.33	2.70
IV				
2S	− 1.42	1.49	2.36	2.83

### Energy alignment of QD and dye orbitals

The bandgaps of the bare Cd_33_Se_33_ QD in vacuum were calculated to be 2.78 eV, which corresponds with experimental data for magic-sized nanocrystals [44, 45]. The functionalization of the Cd_33_Se_33_ QD with N719 dye introduces dye states into the bandgaps of the QD. These mid-gap states reduce the bandgaps of nanocomposites and serve as charge recombination centres for electron transfer, hole transfer, and electron–hole recombination processes, as illustrated in Fig. 1. Among all the structures, the 2S has the smallest bandgaps.

**Fig. 3:**
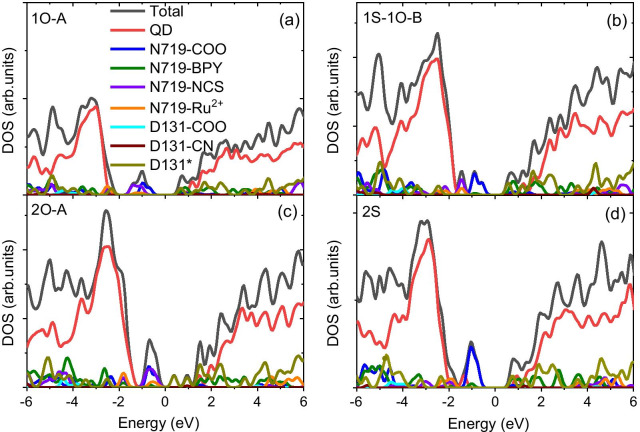
PDOS of the selected binding geometries of N719 dye attached to the Cd_33_Se_33_ QD@D131 dye composite in vacuum. The red, green, olive, violet, orange, cyan, wine, and dark yellow lines represent the contributions of molecular fragments associated with the Cd_33_Se_33_ QD and N719 and D131 dyes. D131* denotes the molecular component from D131 dye, excluding the carboxylate and isocyanate groups

In a vacuum, the highest occupied states are dictated by N719 dye molecules with carboxylate groups as anchors, as shown in Fig. 3a, c and Additional file 1: Fig. S2c, or with mixed carboxylate and isocyanate groups as anchors, as depicted in Fig. 3b and Additional file 1: Fig. S2c, d. When two carboxylate groups from different bipyridine ligands are involved in binding N719 dye to the QD surface, they destabilize the surface states of the QD and introduce localized mid-gap states into the QD, as shown in Additional file 1: Fig. S2b, e, f and Additional file 1: Table S2. Due to unfavourable energy alignments, these mid-gap states disable the hole transfer pathway from the QD to the dye. In addition, they serve as nonradiative recombination centres that promote the blinking of QDs [46, 47]. Such a feature is distinctively different from that of CdSe QDs functionalized with a single dye [11]. In the latter case, the highest occupied state of the Cd_33_Se_33_ QD@N719 dye composite is dictated by the N719 dye when carboxylate groups are involved as anchors [11], creating favourable conditions for the hole transfer from the QD to the dye. Furthermore, the inclusion of isocyanate groups as anchors stabilizes the dye states, which are pushed deep inside the QD VB, disfavouring hole transfer from the QD to the dye in the Cd_33_Se_33_ QD@N719 dye composite. In contrast, isocyanate anchors have no significant impact on the dye states in the N719-D131@Cd_33_Se_33_ composite, as depicted in Fig. 3b and Additional file 1: Fig. S2c, d, which suggests that the co-adsorption of D131 dye weakens the impact of isocyanate anchors on the frontier orbitals of occupied states in QD@dye composites, agreeing with observations from previous experiments [8, 22].

**Fig. 4:**
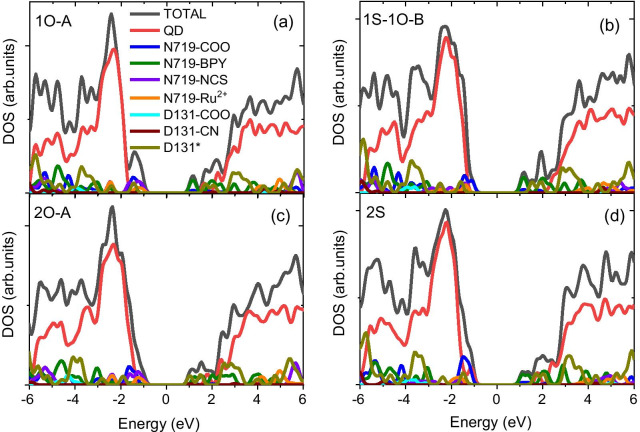
PDOS of the selected binding geometries of N719 dye attached to the Cd_33_Se_33_ QD@D131 dye composite in benzene

The inclusion of a solvent stabilizes the dye states in relation to the QD states, pushing the dye states toward the lower energy levels of the QD VB (Fig. 4; Additional file 1: Fig. S3), due to the screening of dipole–dipole interactions between the QD and the dye [13]. The energy shifts for the structures involving carboxylate anchors are generally smaller than those involving isocyanate anchors due to the slightly smaller dipole moments in these structures [11, 13]. As such, thermodynamic conduction for hole transfer is retained in the structures with carboxylate bridges (1O-A, 1O-B).

In the unoccupied levels, the CB edge of QD@dye composites is dictated by the N719 dye states irrespective of the anchors for all structures in both vacuum and solvent. This suggests that the lowest unoccupied state of the dye is strongly stabilized due to the co-adsorption of D131 dye, which increases the driving force of electron injection from the QD to the dye at the lowest unoccupied levels. Such a feature is notably different from the case of the N719@Cd_33_Se_33_ composite. In the latter case, the character of the CB edge of the composite depends on the specific type of the linkage group and solvent environment. In particular, when carboxylate groups are involved as anchors, the lowest unoccupied state of the composite is dictated by the QD, which disables the electron transfer pathway from the QD to the dye due to unfavourable thermodynamic conduction.

### Electronic coupling

Charge transfer occurs when the donor and acceptor energy levels closely approach one another, where the electron in the donor recombines with the hole in the acceptor as described in Fig. 1. To describe the electron transfer in molecular electronics, three parameters are typically used to determine the charge transfer rate, i.e. the driving force, the reorganization energy, and the electronic coupling [32]. In particular, the electronic coupling shows a strong dependence on geometrical structures, which can be expressed as [48]

2 $$H_{{{\text{DA}}}} = \alpha \exp \left( { - \frac{\beta R}{2}} \right)$$

where *α* and *β* are system-dependent parameters and *R* is the distance between donor and acceptor. The electronic coupling is sensitive to the binding geometry of dye on the QD surface since varying the binding geometries of the dye changes the distance between donor and acceptor, and accordingly changes the electronic coupling.

Table 2 and Additional file 1: Table S4 show the calculated electronic couplings of different charge transfer pathways for all binding configurations of N719-D131@Cd_33_Se_33_ and N719@Cd_33_Se_33_ composites in a vacuum, respectively. Incorporating a solvent is expected to increase the electronic couplings for all binding configurations because the solvation increases the delocalization of electron densities at the occupied and unoccupied energy levels of the QD (Additional file 1: Tables S2 and S3), which increases the electronic couplings accordingly.

**Table 2:** Electronic couplings for different charge transfer pathways (***et***, ***ht***, and ***re***), as depicted in Fig. 1

Binding configuration	Electronic coupling (eV)		
	***et***	***re***	***ht***
I			
2O-A	2.99 × 10^−6^	2.03 × 10^−9^	5.95 × 10^−6^
2O-B	1.50 × 10^−6^	7.85 × 10^−7^	–
2O-C	2.32 × 10^−5^	5.95 × 10^−7^	–
II			
1O-A	4.33 × 10^−4^	5.83 × 10^−10^	7.98 × 10^−6^
1O-B	3.93 × 10^−6^	1.69 × 10^−10^	2.73 × 10^−5^
III			
1S–1O-A	4.33 × 10^−5^	6.42 × 10^−8^	4.99 × 10^−7^
1S–1O-B	5.22 × 10^−5^	5.02 × 10^−10^	3.88 × 10^−7^
2S–1O	4.72 × 10^−5^	1.45 × 10^−7^	3.73 × 10^−7^
2O–1S	5.28 × 10^−6^	1.06 × 10^−6^	2.14 × 10^−5^
IV			
2S	9.15 × 10^−6^	1.48 × 10^−7^	2.56 × 10^−7^

The calculated electronic couplings of ***et*** for structures in Groups I and II of N719-D131@Cd_33_Se_33_ composites are generally smaller than those of N719@Cd_33_Se_33_ composites. The opposite situation is observed for Groups III and IV, that is, the electronic couplings for the ***et*** of Groups III and IV in N719-D131@Cd_33_Se_33_ composites are generally greater than those of N719@Cd_33_Se_33_ composites. This suggests that the co-adsorption of D131 dye increases the electronic couplings of the ***et*** between the N719 dye and CdSe QD when the carboxylate groups are involved as anchors; however, it decreases their couplings at the lowest unoccupied levels when the isocyanate groups are involved as anchors. Such a feature is caused by the interacting distance between the localized electron and hole. As shown in Additional file 1: Table S2, when carboxylate groups are involved as anchors, the electron density of the QD is localized on the side attached to the dye; however, when the isocyanate groups are involved as anchors, the electron density of the QD is far from the side that is attached to the dye.

The calculated values of electronic couplings of ***et*** are greater than those of ***re*** for all structures, suggesting an efficient separation of electron–hole pairs generated in photoexcited QDs due to faster electron transfer than back electron transfer (electron–hole recombination). This is fundamentally different from the case of the N719@Cd_33_Se_33_ composite. In the latter case, the calculated electronic couplings of the ***et*** and ***re*** for all binding configurations show no significant differences between them, suggesting a comparable rate for electron transfer and back electron transfer. Furthermore, for structures in Groups I and II, the calculated values of electronic couplings for ***ht*** are in the same order of magnitude as those for ***et***, suggesting a comparable rate of hole and electron transfers when carboxylate groups are involved as anchors. This is consistent with previous experimental observations that the attachment of the N719 dye increases the photocurrents in CdSe-QD-based solar cells [4, 49]. However, the calculated values of the electronic couplings for ***ht*** are up to two orders of magnitude smaller than those for ***et*** in Groups III and IV, which indicates that the involvement of isocyanate groups as anchors favours ***et*** rather than ***ht***.

The hole extraction is less affected by the size of the QD since increasing the size only affects the energy level of the lowest unoccupied orbital, while the energy level of the highest occupied orbital is barely changed. This is in line with previous theoretical work stating that the VB edge barely changes when increasing the size of CdSe QDs from 1.5 to 2.2 nm, while the CB edge shifts [13].

Ligand passivation was not considered in the current model. Previous studies have shown that ligand loss might contribute to the surface trap states that promote nonradiative recombination [50, 51]. However, the significant surface reconstruction in small Cd_33_Se_33_ QDs removes any surface trap states. As such, the current model can be treated as an idealized model without any surface traps.

Additionally, the Förster resonance energy transfer (FRET) might be computed with the charge transfer due to the overlap of QD and dye frontier orbitals. However, the FRET process typically occurs on a nanosecond timescale compared to the femtosecond timescale of charge transfer. The FRET process is associated with the dipole–dipole interactions, while the charge transfer is associated with the interactions of frontier orbitals. The latter is generally stronger than the former; therefore, the charge transfer dominates the energy conversion efficiency of QD@dye composites.

## Conclusion

In the present work, we performed DFT-based calculations to study the effect of a co-adsorbent, D131 dye, on the charge transfer properties, that is, energy alignments and electronic couplings, of a CdSe QD functionalized with N719 dye. The energy alignments between the CdSe QD and N719 dye are sensitive to the specific type of linkage groups. In particular, when one carboxylate group is involved as the anchor, the energy of the highest occupied state of the N719 dye is higher than that of the CdSe QD, creating a favourable condition for hole transfer from the QD to the dye. However, the involvement of two carboxylate anchors, which provide the most stable adsorption configuration of the dye onto the QD, creates the mid-gap states from the QD inside the bandgap of the composites, disabling the hole transfer pathway and promoting nonradiative recombination. Furthermore, the involvement of isocyanate anchors reduces the binding stability of the dye onto the QD surface and stabilizes the dye states, which are pushed deep inside the QD VB.

The calculated electronic couplings showed a comparable rate for the electron and hole transfers in those structures involving only carboxylate anchors, which also possess larger values of electronic couplings for electron transfer than for electron back transfer. However, the involvement of isocyanate anchors in those structures means that the electron transfer and hole transfer are no longer on an equal footing, with the former being favoured. The current calculations only allow for an estimation of the charge transfer by analysing the energy alignments and electronic couplings of different charge transfer pathways. A complete picture of the charge transfer process should include the electron–phonon coupling, electron–hole coupling, and reorganization energies of solvent and nuclear configurations. Despite the lack of these dynamic properties, the current study allows for an evaluation of the extent to which charge transfer processes depend on the dye-binding configurations when coupled to a QD surface.

## Supplementary Information

**Additional file 1.** Projected density of states and frontier molecular orbitals for QD@dye composites.

## Data Availability

All data generated or analysed during this study are included in this published article.

## Abbreviations

QD: Quantum dot
DFT: Density functional theory
VB: Valence band
CB: Conduction band
*et*: Electron transfer
*ht*: Hole transfer
*re*: Electron–hole recombination
CDFT: Constrained density functional theory
N719: Di-tetrabutylammonium cis-bis(isothiocyanato)bis(2,2′-bipyridyl-4,4′-dicarboxylato)ruthenium(II)
D131: (2*E*)-2-Cyano-3-{4-[4-(2,2-diphenylvinyl)phenyl]-1,2,3,3a,4,8b-hexahydrocyclopenta[b]indol-7-yl}acrylic acid
MLCT: Metal-to-ligand charge transfer
CdSe: Cadmium selenium
CdTe: Cadmium telluride
TDOS: Total density of states
PDOS: Projected density of states
O: Oxygen
S: Sulphur
