# Supplementary material for: Effects of co-adsorption on interfacial charge transfer in a quantum dot@dye composite
Source: Nanoscale Res Lett. 2021 Sep 20;16:147. doi: 10.1186/s11671-021-03604-0 (PMC8452815; doi:10.1186/s11671-021-03604-0)
Supplement: Supplementary file 1 — Additional file 1. Projected density of states and frontier molecular orbitals for QD@dye composites. [file 11671_2021_3604_MOESM1_ESM.docx]

| I | II | III |
| --- | --- | --- |
| 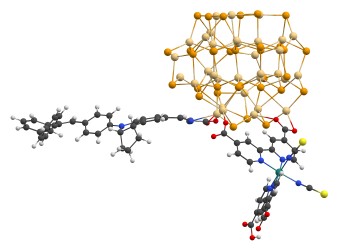  2O-A  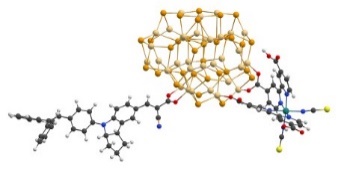  2O-C | 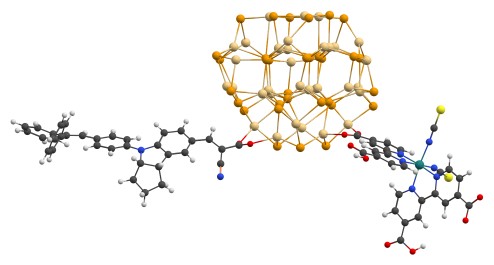  1O-B | 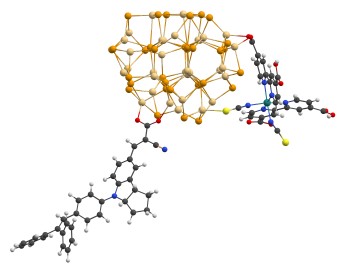  1S-1O-B  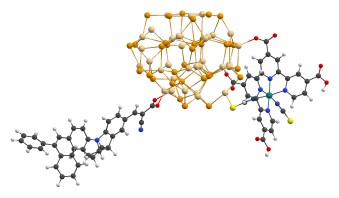  2O-1S  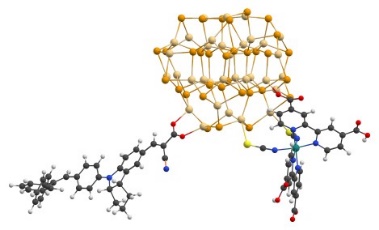  2S-1O |

**Figure S1.** Representative geometries of bare Cd_33_Se_33_ QD and N719-D131@Cd_33_Se_33_ composite. The geometry optimization was performed in vacuum.


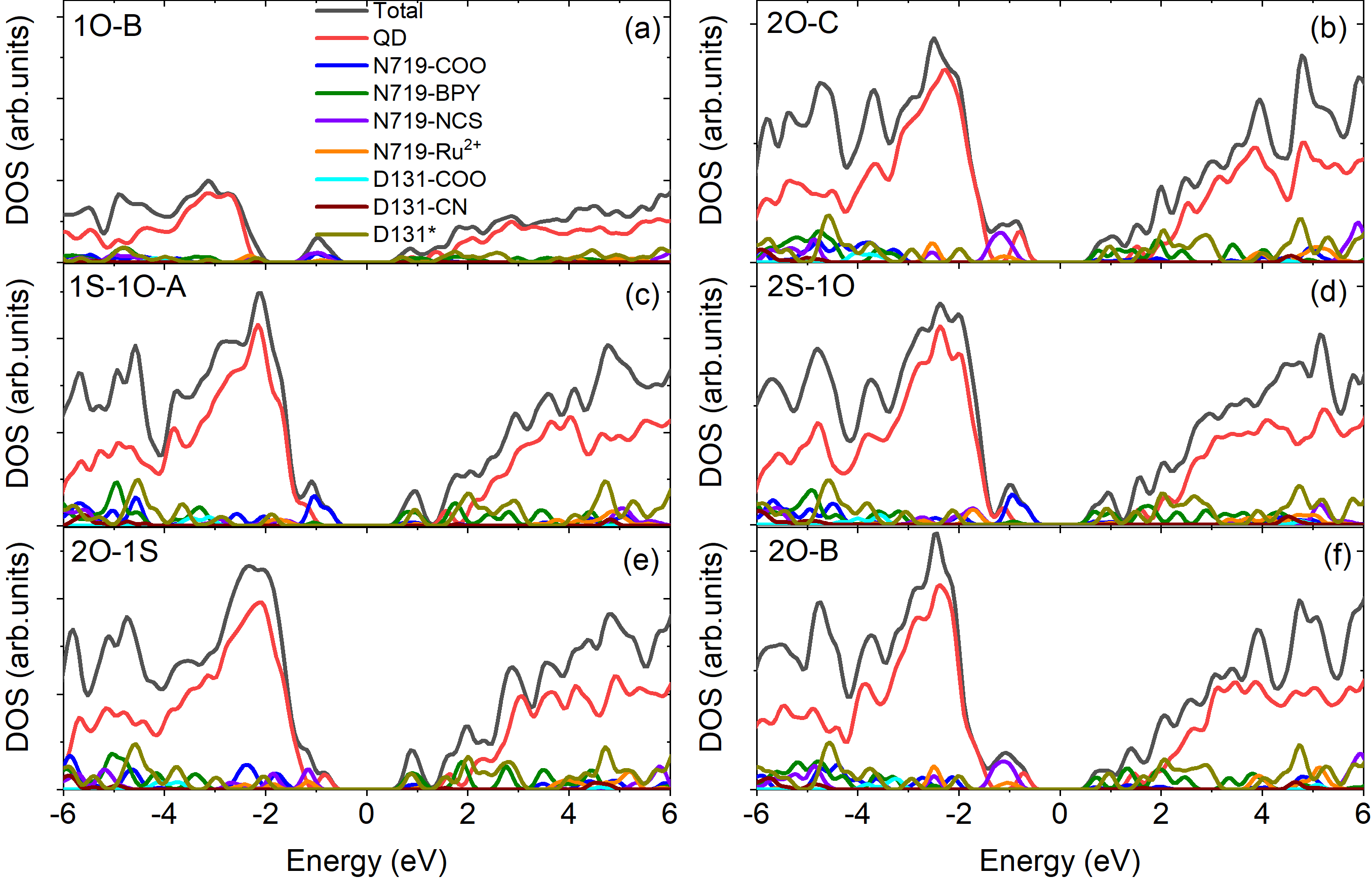


**Figure S2.** PDOS of the selected binding geometries of N719 dye attached to Cd_33_Se_33_ QD@D131 composite in vacuum. The red, green, olive, violet, orange, cyan, wine and dark yellow lines represent the contributions of molecular fragments associated with the Cd_33_Se_33_ QD, N719 and D131 dyes. D131* denotes the molecular component from D131 dye excluding the carboxylate and cyanide groups.


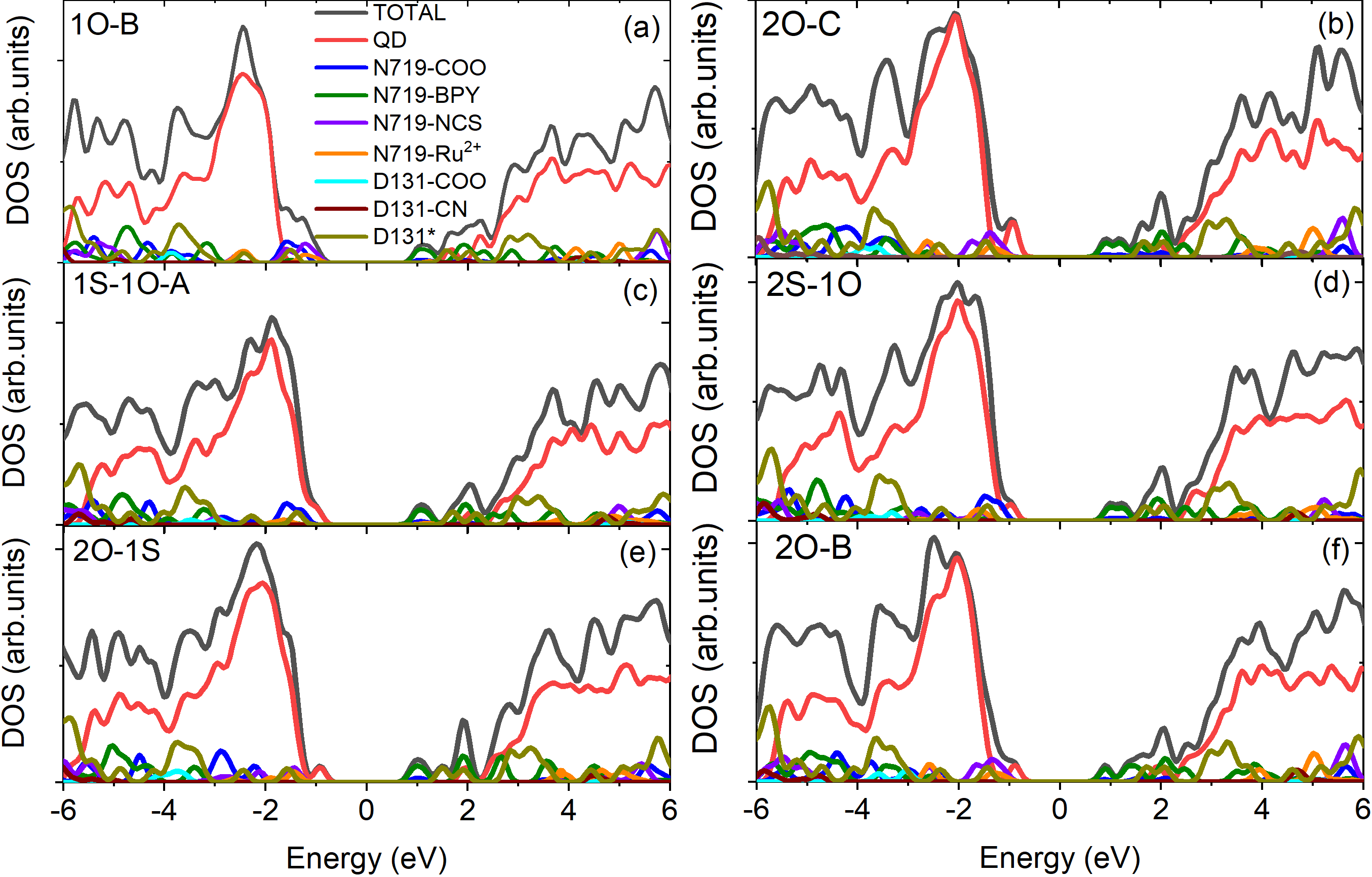


**Figure S3.** PDOS of the different binding geometries of N719 dye attached to Cd_33_Se_33_ QD@D131 composite in benzene.

| **Table S1.** Electronic and geometrical properties of the N719@Cd_33_Se_33_ composite. The geometries were optimized in vacuum.^1^   \|  \| Binding configuration \| binding energy (eV) \| band gap  (eV) \| Cd-O bond (Å) \| Cd-S bond (Å) \| \| --- \| --- \| --- \| --- \| --- \| --- \| \| I \| 2O-A \| -5.78 \| 1.65 \| 2.27 \|  \| \| 2O-B \| -5.65 \| 1.44 \| 2.27 \|  \| \| 2O-C \| -5.39 \| 1.48 \| 2.28 \|  \| \| II \| 1O-A \| -3.25 \| 1.67 \| 2.28 \|  \| \| 1O-B \| -3.33 \| 1.61 \| 2.28 \|  \| \| III \| 1S-2O \| -4.41 \| 1.97 \| 2.21 \| 2.75 \| \| 2S-1O \| -3.14 \| 1.15 \| 2.17 \| 2.89 \| \| 1S-1O-A \| -2.85 \| 1.74 \| 2.18 \| 2.74 \| \| 1S-1O-B \| -3.29 \| 1.83 \| 2.32 \| 2.75 \| \| IV \| 2S \| -1.22 \| 1.23 \|  \| 2.88 \|   **Table S2.** Frontier molecular orbitals of N719-D131@Cd_33_Se_33_ composite in vacuum. | | | | | |
| --- | --- | --- | --- | --- | --- | --- | --- | --- | --- | --- | --- | --- | --- | --- | --- | --- | --- | --- | --- | --- | --- | --- | --- | --- | --- | --- | --- | --- | --- | --- | --- | --- | --- | --- | --- | --- | --- | --- | --- | --- | --- | --- | --- | --- | --- | --- | --- | --- | --- | --- | --- | --- | --- | --- | --- | --- | --- | --- | --- | --- | --- | --- | --- | --- | --- |
| MOs | I | | | II | |
|  | 2O-A | 2O-B | 2O-C | 1O-A | 1O-B |
| HOMO-5 | 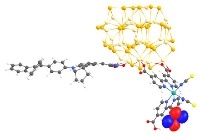 | 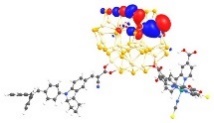 | 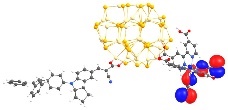 | 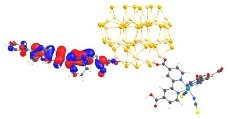 | 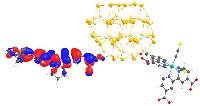 |
| HOMO-4 | 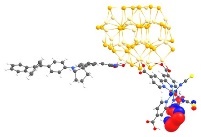 | 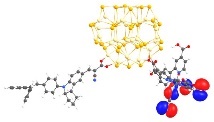 | 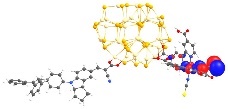 | 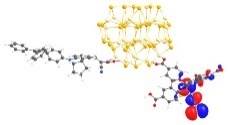 | 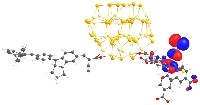 |
| HOMO-3 | 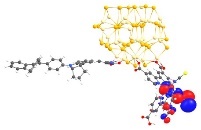 | 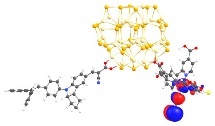 | 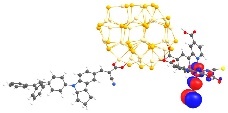 | 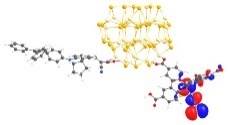 | 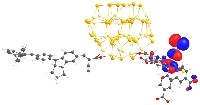 |
| HOMO-2 | 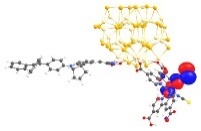 | 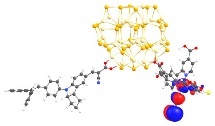 | 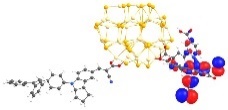 | 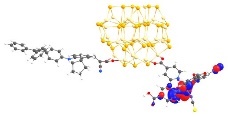 | 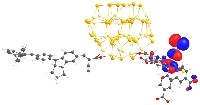 |
| HOMO-1 | 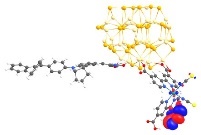 | 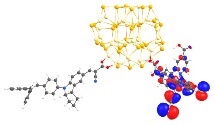 | 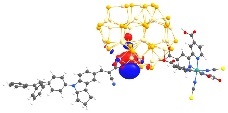 | 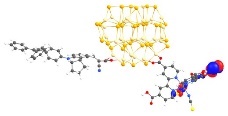 | 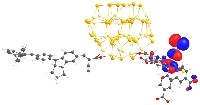 |
| HOMO | 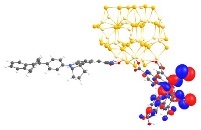 | 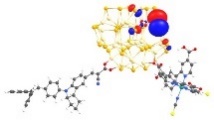 | 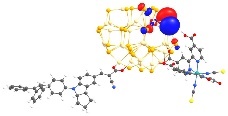 | 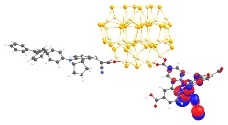 | 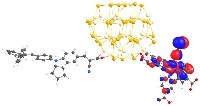 |
| LUMO | 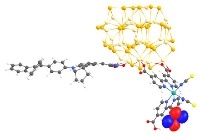 | 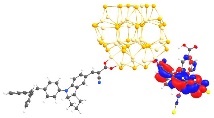 | 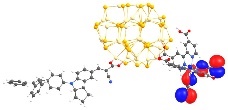 | 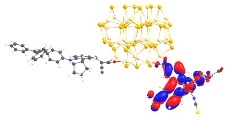 | 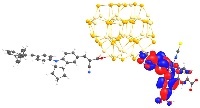 |
| LUMO+1 | 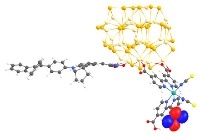 | 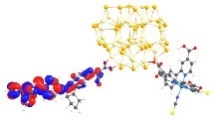 | 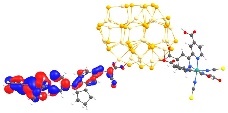 | 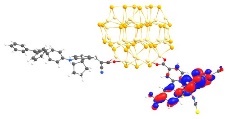 | 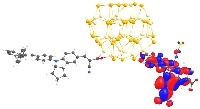 |
| LUMO+2 | 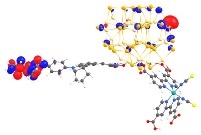 | 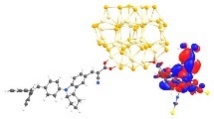 | 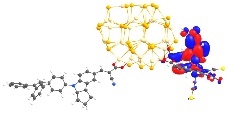 | 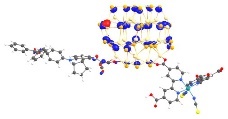 | 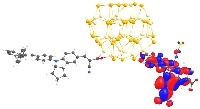 |
| LUMO+3 | 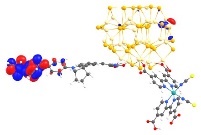 | 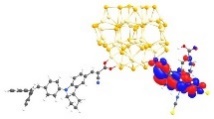 | 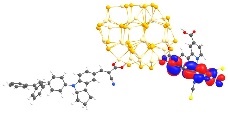 | 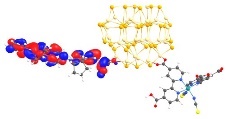 | 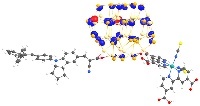 |
| LUMO+4 | 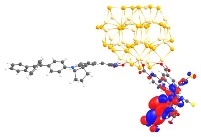 | 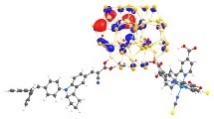 | 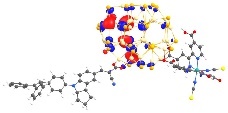 | 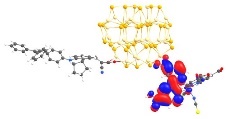 | 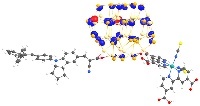 |
| LUMO+5 | 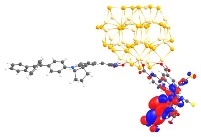 | 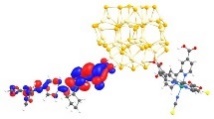 | 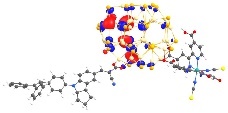 | 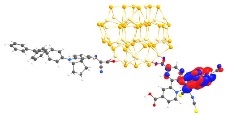 | 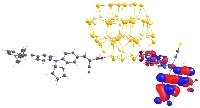 |
| MOs | **III** | | | | **IV** |
|  | 2S-1O | 2O-1S | 1S-1O-A | 1S-1O-B | 2S |
| HOMO-5 | 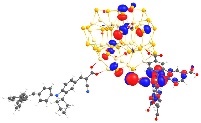 | 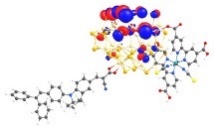 | 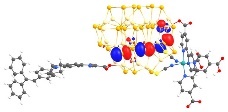 | 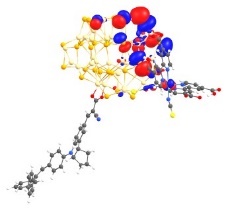 | 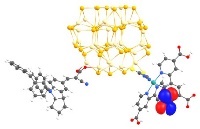 |
| HOMO-4 | 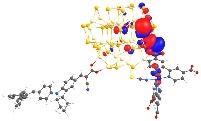 | 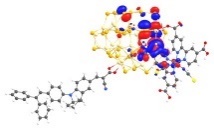 | 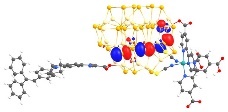 | 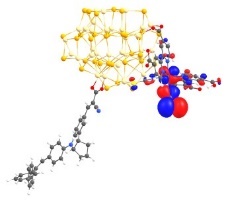 | 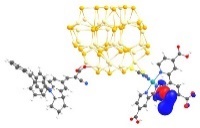 |
| HOMO-3 | 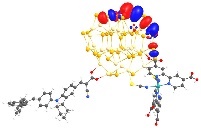 | 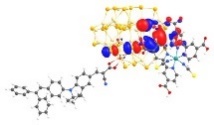 | 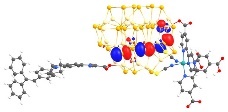 | 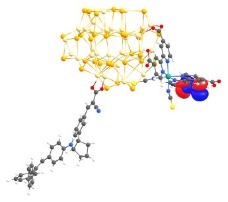 | 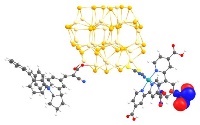 |
| HOMO-2 | 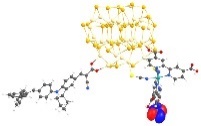 | 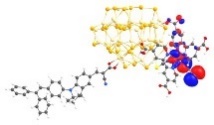 | 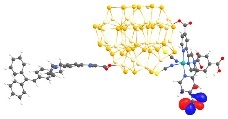 | 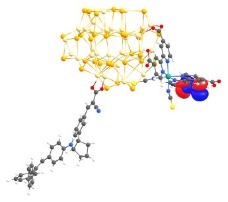 | 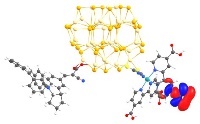 |
| HOMO-1 | 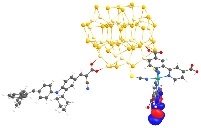 | 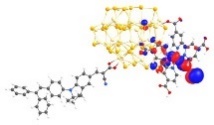 | 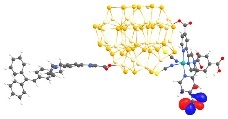 | 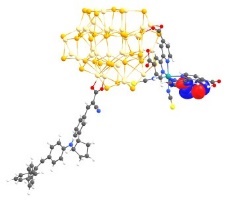 | 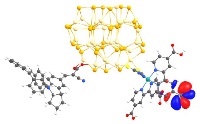 |
| HOMO | 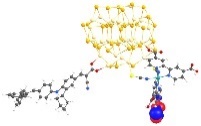 | 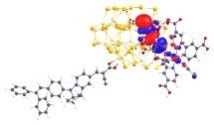 | 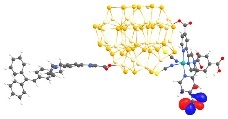 | 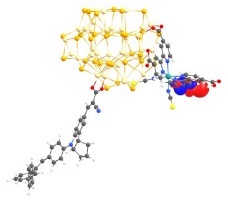 | 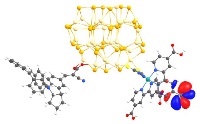 |
| LUMO | 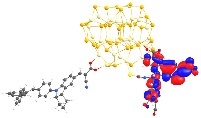 | 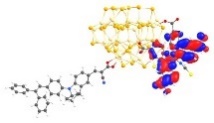 |  |  |  |
| LUMO+1 |  |  |  |  |  |
| LUMO+2 |  |  |  |  |  |
| LUMO+3 |  |  |  |  |  |
| LUMO+4 |  |  |  |  |  |
| LUMO+5 |  |  |  |  |  |

| **Table S3.** Frontier molecular orbitals of N719-D131@Cd_33_Se_33_ composite in benzene | | | | | |
| --- | --- | --- | --- | --- | --- |
| MOs | **I** | | | **II** | |
|  | 2O-A | 2O-B | 2O-C | 1O-A | 1O-B |
| HOMO-5 |  |  |  |  |  |
| HOMO-4 |  |  |  |  |  |
| HOMO-3 |  |  |  |  |  |
| HOMO-2 |  |  |  |  |  |
| HOMO-1 |  |  |  |  |  |
| HOMO |  |  |  |  |  |
| LUMO |  |  |  |  |  |
| LUMO+1 |  |  |  |  |  |
| LUMO+2 |  |  |  |  |  |
| LUMO+3 |  |  |  |  |  |
| LUMO+4 |  |  |  |  |  |
| LUMO+5 |  |  |  |  |  |
| MOs | **III** | | | | **IV** |
|  | 2S-1O | 2O-1S | 1S-1O-A | 1S-1O-B | 2S |
| HOMO-5 |  |  |  |  |  |
| HOMO-4 |  |  |  |  |  |
| HOMO-3 |  |  |  |  |  |
| HOMO-2 |  |  |  |  |  |
| HOMO-1 |  |  |  |  |  |
| HOMO |  |  |  |  |  |
| LUMO |  |  |  |  |  |
| LUMO+1 |  |  |  |  |  |
| LUMO+2 |  |  |  |  |  |
| LUMO+3 |  |  |  |  |  |
| LUMO+4 |  |  |  |  |  |
| LUMO+5 |  |  |  |  |  |

**Table S4.** Electronic couplings for different charge transfer pathways (***et***, ***ht*** and ***re***) in N719@Cd_33_Se_33_ composite.^1^

| Binding configuration | | Electronic coupling (eV) | |
| --- | --- | --- | --- |
|  |  | ***et*** | ***re*** |
| I | **2O-A** | 1.29×10^-6^ | 2.32×10^-7^ |
|  | **2O-B** | 3.60×10^-8^ | 1.84×10^-8^ |
| II | **2O-C** | 8.28×10^-7^ | 1.73×10^-8^ |
|  | **1O-A** | 3.84×10^-8^ | 3.57×10^-8^ |
|  | **1O-B** | 2.29×10^-6^ | 4.20×10^-9^ |
| III | **1S-1O-A** | 8.08×10^-8^ | 1.92×10^-5^ |
|  | **1S-1O-B** | 3.41×10^-4^ | 1.32×10^-2^ |
|  | **2S-1O** | 4.60×10^-4^ | 2.09×10^-2^ |
|  | **2O-1S** | 3.22×10^-6^ | 1.78×10^-6^ |
| IV | **2S** | 7.30×10^-2^ | 1.36×10^-2^ |

1. Cui, P.; Tamukong, P. K.; Kilina, S. J. A. A. N. M., Effect of Binding Geometry on Charge Transfer in CdSe Nanocrystals Functionalized by N719 Dyes to Tune Energy Conversion Efficiency. **2018,** *1* (7), 3174-3185.
